# Deletion of Glutamate Delta-1 Receptor in Mouse Leads to Enhanced Working Memory and Deficit in Fear Conditioning

**DOI:** 10.1371/journal.pone.0060785

**Published:** 2013-04-03

**Authors:** Roopali Yadav, Brandon G. Hillman, Subhash C. Gupta, Pratyush Suryavanshi, Jay M. Bhatt, Ratnamala Pavuluri, Dustin J. Stairs, Shashank M. Dravid

**Affiliations:** 1 Department of Pharmacology, Creighton University, Omaha, Nebraska, United States of America; 2 Department of Psychology, Creighton University, Omaha, Nebraska, United States of America; University of Victoria, Canada

## Abstract

Glutamate delta-1 (GluD1) receptors are expressed throughout the forebrain during development with high levels in the hippocampus during adulthood. We have recently shown that deletion of GluD1 receptor results in aberrant emotional and social behaviors such as hyperaggression and depression-like behaviors and social interaction deficits. Additionally, abnormal expression of synaptic proteins was observed in amygdala and prefrontal cortex of GluD1 knockout mice (GluD1 KO). However the role of GluD1 in learning and memory paradigms remains unknown. In the present study we evaluated GluD1 KO in learning and memory tests. In the eight-arm radial maze GluD1 KO mice committed fewer working memory errors compared to wildtype mice but had normal reference memory. Enhanced working memory in GluD1 KO was also evident by greater percent alternation in the spontaneous Y-maze test. No difference was observed in object recognition memory in the GluD1 KO mice. In the Morris water maze test GluD1 KO mice showed no difference in acquisition but had longer latency to find the platform in the reversal learning task. GluD1 KO mice showed a deficit in contextual and cue fear conditioning but had normal latent inhibition. The deficit in contextual fear conditioning was reversed by D-Cycloserine (DCS) treatment. GluD1 KO mice were also found to be more sensitive to foot-shock compared to wildtype. We further studied molecular changes in the hippocampus, where we found lower levels of GluA1, GluA2 and GluK2 subunits while a contrasting higher level of GluN2B in GluD1 KO. Additionally, we found higher postsynaptic density protein 95 (PSD95) and lower glutamate decarboxylase 67 (GAD67) expression in GluD1 KO. We propose that GluD1 is crucial for normal functioning of synapses and absence of GluD1 leads to specific abnormalities in learning and memory. These findings provide novel insights into the role of GluD1 receptors in the central nervous system.

## Introduction

The delta family of ionotropic glutamate receptors (iGluRs) has two members glutamate delta 1 (GluD1) and glutamate delta 2 (GluD2) receptors, which share ∼60% homology between themselves. Both GluD1 and GluD2 receptors do not exhibit typical agonist induced ion channel currents like other ionotropic glutamate receptors [1]. GluD1 is expressed throughout the forebrain during development and shows highest expression in the hippocampus in the adult rodent brain [2], [3]. Genetic association and genome wide association studies indicate that the dysregulation of GluD1 may play a role in neuropsychiatric conditions such as schizophrenia, bipolar disorder, major depressive disorder and autism spectrum disorders (ASDs) [4], [5], [6], [7], [8], [9], [10], [11], [12], [13], [14], [15]. Although physiology of GluD2 receptors expressed at the parallel fiber-Purkinje cell synapse is well established, the function of the GluD1 receptor in the central nervous system remains poorly understood. We recently demonstrated for the first time that GluD1 knockout mice (GluD1 KO) exhibit deficits in emotional and social behaviors [16]. These behaviors were highlighted by lower anxiety-like behavior, hyperaggression, higher depression-like behavior and deficits in social interaction. This behavioral phenotype in GluD1 KO mimics certain features of GluD1 associated neuropsychiatric disorders. Many of the GRID1 associated neuropsychiatric disorders such as schizophrenia and ASDs are also associated with unique cognitive deficits. A previous study has demonstrated that GluD1 KO show no deficit in spatial learning in a Morris water maze test [2] however it is not fully understood whether GluD1 deletion may affect other forms of learning and memory.

In the present study we tested GluD1 KO in a battery of behavioral tests for learning and memory including tests for short-term working memory, long-term reference memory, reversal learning as well as associative learning. We found that GluD1 KO have enhanced working memory in the radial maze and Y-maze alternation test, a deficit in reversal learning in the Morris water maze test and a deficit in contextual and cue fear conditioning. The deficit in contextual fear acquisition was reversed by D-Cycloserine (DCS). We have previously demonstrated that GluD1 deletion leads to abnormalities in synaptic proteins in the prefrontal cortex and amygdala [16]. Here we show that abnormalities in synaptic proteins are also observed in the hippocampus due to deletion of GluD1. Some of these abnormalities such as lower AMPA receptor expression is similar to that observed in the prefrontal cortex. Additionally, deletion of GluD1 leads to an increase in PSD95 expression and a reduction in GAD67 expression. Together with our previous findings we propose that GluD1 receptor is essential for normal synapse formation and maintenance and deletion of GluD1 leads to synaptic abnormalities in the amygdala, prefrontal cortex and hippocampus that lead to social and emotional deficits as well as deficits in learning and memory.

## Materials and Methods

### Ethics statement

All experimental protocols were approved by the Creighton University Institutional Animal Care and Use Committee Policies and Procedures. In this study strict measures were taken to minimize pain and suffering to animals in accordance with the recommendations in the Guide for Care and Use of Laboratory Animals of the National Institutes of Health. The IACUC protocols for these studies were 0893, 0865 and 0862.

### Generation and genotyping of GluD1 knockout mice

GluD1 KO mice were obtained from Dr. Jian Zuo, St. Jude’s Children’s Hospital [2]. These mice had been generated by creating a targeting construct that deleted exons 11 and 12 of the GluD1 gene (GRID1). The targeted disruption ensured removal of three of the four transmembrane domains of the GluD1 receptor and introduced a frameshift after exon 12. In the PCR analysis no 220 bp wildtype bands (in the deleted region) were detected in the homozygous GluD1 KO mice. All mice analyzed were from a mixed background of 129/SvEv and C57BL/6 [2]. Genotyping was done as previously described [2]. The primers used for the reaction were as follows: a pair of primers from the deleted region of GluD1; 5′GCAAGCGCTACATGGACTAC 3′ and 5′GGCACTGTGCAGGGTGGCAG 3′ and a pair of primers from the targeting vector; 5′CCTGAATGAACTGCAGGACG 3′ and 5′CGCTATGTCCTGATAGCGATC 3′.

### Mouse husbandry

Wildtype (WT) and GluD1 KO male mice, aged 8 weeks were group housed (4–5 mice) in the animal house facility at a constant temperature (22±1°C) and a 12-hr light-dark cycle with free access to food and water. Behavioral testing was performed between 9:00 a.m. and 4:00 p.m. The study did not involve using female mice to avoid the confounding effects of the estrus cycle on behavioral and neurochemical measures. The WT and GluD1 KO mice littermates for behavioral studies were obtained from previously genotyped parent cages.

### Behavioral testing

As per the requirements of the tests, mice were handled for 3 days to acclimate them to the experimenter before subjecting them to the experimental procedures. All experimental mice were placed in the experimental room at least 60 min before beginning any behavioral protocol. Unless indicated otherwise, all experimental environments were thoroughly cleaned with 70% ethanol between trials and allowed to dry. All behavioral procedures were video-recorded and scored by an individual blind to the genotype of the mouse via a random coding system of the video files.

### D-Cycloserine treatment

There were four groups of mice, WT saline, GluD1 KO saline, WT DCS and GluD1 KO DCS. DCS (Sigma-Aldrich (C6880); St. Louis, MO, USA) was dissolved in 0.9% saline. Freshly dissolved DCS was used for experiments. Mice were administered a single dose of 30 mg/kg DCS or saline (volume of 80–100 µl), 30 minutes prior to the beginning of contextual fear conditioning. The dose of DCS used was decided based on similar studies [17], [18].

### Eight arm radial maze

Working and reference memory were tested with an eight arm radial maze (Coulbourn Instruments, Whitehall, PA, USA) and took place over an 11-day session broken into three phases; acclimation, training and testing as previously described [19]. During all phases of the procedure animals had 23 hrs of food deprivation to increase saliency of food pellets (45 mg pellets; BioServ, Frenchtown, NJ, USA) located at the end of each baited arm. During acclimation (days 1–3) animals were allowed to explore the eight arm radial maze with randomly placed food pellets throughout the maze. During training (days 4–6) animals were placed in the maze facing arm 1 with arms 1, 2, 4 and 7 baited with a food pellet at the end of each arm and with arms 3, 5, 6 and 8 closed. Four training sessions occurred for each animal on each of the training days which were days 4–6, each lasting until all food pellets had been retrieved or 5 min had elapsed. The testing phase occurred over days 7–11. During testing all arms of the radial maze were open. Four testing sessions occurred each day for every animal and lasted until all food reward was retrieved or 5 min had elapsed. Entries into unbaited arms were counted as reference memory errors and re-entries into previously baited arms were counted as working memory errors. Errors were compiled daily for each animal to derive average errors per day.

### Y-maze spontaneous alternation

Y-maze test was conducted similar to as previously described [20]. A Y maze with three identical arms of plexiglass (40×4.5×12 cm) 120° apart was placed in the center of a room. The walls of each arm had distinct design that provided visual cues. Each mouse was placed at the end of one arm facing the center and allowed to explore the maze for a period of 8 minutes. Sessions were video recorded and scored for entries into arms. A mouse was excluded from further analysis if it did not have any new entries for a period of more than 2 min or had less than a total of 12 arm entries during the 8 min period. Alternation behavior was defined as consecutive entries into each of the arms without repetition. Percent of spontaneous alternation was calculated as number of actual alternations divided by possible alternations (total arm entries-2) X 100.

### Novel object recognition

The novel object recognition chamber was a square open field (25.4×25.4×17.8 cm). The novel object recognition task was performed in three phases; environmental acclimation, training and testing as previously described [19]. During acclimation animals were handled 1–2 min a day for 3 days. On days 4 and 5 of training mice were placed in the chamber with two identical objects and allowed to explore for 10 min. Twenty-four hours after training on day 6 one familiar object was exchanged with a novel object and mice were allowed to explore the experimental apparatus for 10 min. Location of the novel object was counterbalanced. Percent time spent around the novel object was recorded.

### Morris water maze

Mice were tested for spatial learning in the Morris water maze test. The Morris water maze tank used was 107 cm diameter circular tank with 56 cm high walls filled half-way with water (26±1°C) to a height of 28 cm that was made opaque with a non-toxic white paint. The inside of the tank was painted white and was non-reflective. Visual cues were placed on the walls of the room and also on the walls of the circular tank. The escape platform used for the test was made with PVC. The top of the platform was 10 cm in diameter and had ridges to allow for a firm grip. The platform was submerged 1 cm below the water surface on test days. A PVC pipe was used as the vertical post for the platform, the base was a larger piece of PVC with gravel filled inside to provide stability when mice land on the top of the platform [21]. The test was conducted as previously described by [22] with minor modifications. The experiment consisted of four phases, stationary visible platform task, acquisition learning, probe trial for acquisition learning, reversal learning, probe trial for reversal learning. Day 1 was the stationary visible platform task, for this the escape platform was placed 0.5 cm above the water level in the tank and was 30 cm away from the edge of the quadrant in which it was positioned. A 10 cm vertical pole with a flag on top was placed on the platform. Mice were released from one of the four positions at the pool periphery. In order to escape mice had to swim to the platform. Each mouse was subjected to four trials with one hour inter-trial intervals. On days 2 to 6 the hidden platform task was conducted to test acquisition learning. Four trials with one hour inter-trial intervals were conducted on each of the acquisition learning days. The water level was adjusted so that the escape platform was submerged approximately 1 cm below the water surface. Mice were allowed to search the platform for 90 sec. Mice finding the escape platform were left on it for 15 sec while unsuccessful mice were assigned a 90 sec latency and placed on the platform for 15 sec. A single probe trial was performed on day 7 during which the escape platform was removed from the pool and mice were released from the point furthest from the former platform location, the time spent in the respective quadrants was recorded for 60 sec. On days 8, 9 and 10, the reversal learning task was conducted. For the reversal learning task the escape platform was moved to the opposite quadrant relative to its position during acquisition learning. The reversal learning task was performed similar to acquisition learning with four trials per day with one hour inter-trial intervals. On day 11 a single reversal probe trial was conducted. Indirect lighting was used in the room where the maze was kept. The experimenter moved out of the room after releasing the mice into the tank. Latency to find the platform was scored and plotted.

### Fear conditioning

Fear conditioning was done as previously described by [19]. Briefly, for fear conditioning, mice were placed in a Plexiglas rodent conditioning chamber (chamber A; model 2325-0241 San Diego Instruments, San Diego, CA, USA) with a metal grid floor that was enclosed in a sound-attenuating chamber. The chamber was illuminated with either red or white light depending on the type of conditioned stimulus (CS, tone or light) associated with the unconditioned stimulus (US, foot-shock). Chamber A was cleaned with a 19.5% ethanol, 1% vanilla solution to give the chamber a distinct scent. For CS testing; mice were placed in a novel Plexiglas chamber (chamber B; model 2325-0241 San Diego Instruments, San Diego, CA, USA) with different visual cues and a solid Plexiglas floor to minimize generalization to the conditioning chamber. Chamber B was cleaned with a 70% ethanol solution complimented with linen-scented air freshener and illuminated with a 40 watt white light unless indicated otherwise. White noise was provided in each isolation cabinet with a fan. A web-camera (Logitech QuickCam, Fremont, CA, USA) was mounted at the top of each isolation chamber to videotape all sessions.

### Contextual fear conditioning

In order to determine the contextual influence on fear conditioning, we conducted the contextual fear conditioning test in wildtype and GluD1 KO mice. Fear conditioning was performed in chamber A described above. Animals were not pre-exposed to chamber A prior to conditioning. On the day of conditioning (day 1) mice were placed in chamber A for 3 min followed by three presentations of the US (foot-shock) with a 90 sec inter trial interval (ITI). The US was a 0.8 mA foot-shock delivered for 2 sec. Mice were removed from chamber A 2 min after the final US presentation. On test day (day 2), the mice were placed in chamber A for 4 min and freezing behavior was recorded. Freezing behavior prior-to and after presentation of the US on day 1 and during testing on day 2 was recorded as absence of all non-respiratory movements every five seconds. Scores of 0 for immobility and 1 for movement were averaged and divided by the total number of readings to derive a percent freezing. Behavioral freezing was also analyzed with the Freeze Monitor System (San Diego Instruments, San Diego, CA, USA) software to verify visual scores.

### Light-cue conditioning

For the light-cue conditioning prior to conditioning (day 0) animals were acclimated to chamber A for 30 min. On the day of conditioning (day 1) mice were placed in chamber A for 3 min followed by three CS–US pairings. The CS was a white light delivered for 30 sec with a 1 min inter-trial interval (ITI). The US was a 0.8 mA foot-shock delivered for 2 sec that co-terminated with the CS. Mice were removed from chamber A 1 min after the final CS–US pairing. On testing day (day 2) mice were placed in chamber B and after a 2 min delay exposed to the CS for 2 min and removed from the chamber 2 min later. The houselight in chambers A and B were provided by a 25 watt red light. Behavioral freezing was scored as described earlier in the methods.

### Tone-cue conditioning

The fear conditioning was same as light cue conditioning except that the CS was an 85 dB, 3 kHz tone delivered for 30 sec with a 1 min inter-trial interval (ITI). This same CS was used during post-test 24 hrs after conditioning.

### Tone + Light conditioning

Fear conditioning was performed similar to the light cue fear conditioning except that the CS was an 85 dB, 3 kHz tone delivered for 30 sec with a 1 min inter-trial interval (ITI) along with a white light. This same CS was used during post-test 24 hrs after conditioning. House light in chambers A and B was provided by a 25 watt red light.

### Latent inhibition

Latent inhibition was performed in the conditioning apparatus described above. A protocol similar to [23] was followed. WT and GluD1 KO mice were subdivided into two groups; pre-exposed and non-pre-exposed. Two minutes after initial presentation to chamber A pre-exposed animals received 20×10 sec tone presentations with 20 sec intervals on day 1 and 15×10 sec tone with 20 sec intervals on day 2 whereas non-pre-exposed animals were placed in chamber A for the same duration without presentation of the tone. The tone for the pre-exposed groups was an 85 dB, 3 kHz tone. Mice in both groups were then presented with 5×10 sec tone that co-terminated with a 2 sec 0.8 mA foot-shock. On day 3 animals in both groups were placed in chamber B, a contextually different setting, and presented with the tone for two minutes, two minutes after initial cage movement was observed. Level of fear associated with the tone was assessed by scoring level of freezing (immobility) during presentation of the tone in the novel context.

### Pain sensitivity

Pain sensitivity to foot-shock was assessed in the fear conditioning apparatus similar to [19]. Following 2 min of habituation, a series of 2 sec foot-shocks were delivered ranging from 0.1 to 0.8 mA at 0.1 mA ascending increments with 20 sec ITI. All sessions were videotaped and the current intensity required to elicit flinching, vocalization, and jumping behaviors were scored.

### Synaptoneurosome preparation and western blot analysis

For synaptoneurosomal preparation 45–50 day old naive WT and GluD1 KO mice were anesthetized using isoflurane anesthesia, mice were then decapitated and thereafter all experimental procedures were conducted on ice. The hippocampus was crudely dissected out and put into synaptoneurosomal buffer at 4 °C. Thereafter, the fresh tissue, the hippocampi were used for synaptoneurosome preparation and western blotting.

The freshly isolated hippocampi from WT and GluD1 KO mice were homogenized in synaptoneurosome buffer (10 mM HEPES, 1 mM EDTA, 2 mM EGTA, 0.5 mM DTT, 10 µg/ml leupeptin, and 50 µg/ml soybean trypsin inhibitor, pH 7.0) as previously described [24], additionally containing 5 mg/ml pepstatin, 50 mg/ml aprotonin and 0.5 mM phenylmethanesulfonylfluoride (PMSF). From this step forward the homogenate was kept ice-cold at all times to minimize proteolysis throughout the isolation procedure. The homogenate was diluted further with the same volume of synaptoneurosome buffer and briefly and gently sonicated delivering 3 pulses using an output power of 1 Sonic dismembrator Model 100 (Fischer Scientific, NJ, USA). The sample was loaded into a 1.0 ml Luer-lock syringe (BD syringes) and filtered twice through three layers of a pre-wetted 100 µm pore nylon filter CMN-0105-D (Small Parts Inc., Logansport, IN, USA) held in a 13 mm diameter filter holder XX3001200 (Milipore, MA, USA). The resulting filtrate was loaded into a 1 ml Luer-lock syringe and filtered through a pre-wetted 5 µm pore hydrophilic filter CMN-0005-D (Small Parts Inc., Logansport, IN, USA) held in a 13 mm diameter filter holder. The resulting filtrate was centrifuged at 1000 X g for 10 min. The pellet obtained corresponded to the synaptoneurosome fraction. Isolated synaptoneurosomes were resuspended in 75 µl of buffer solution containing 0.32 M sucrose, and 1 mM NaHCO_3_ (pH 7.0).

For western blotting synaptoneurosomes prepared from 45–50 day old naive WT and GluD1 KO mice were loaded on 10% Sodium dodecyl sulfate gel in equal amount (15 µg / well). The samples were run at 114 volts for a duration of 1 hr. Gels were transferred to nitrocellulose membrane (GE Healthcare, Piscataway, NJ, USA), a wet transfer was carried out. The voltage for transfer was kept at 114 volts and duration for which transfer was carried out was 1 hr 15 min. Electrophoresis and transfer apparatuses used were the Biorad mini protean tetra cell (Bio-Rad Laboratories, Inc., Hercules, California, USA). Transfer was followed by blocking with 5% milk in Tris-buffered Saline with 1% Tween 20 (TBST) for 1 hr at room temperature. The primary antibodies; GluA1 (Millipore, Billerica, MA, USA), 1∶1500; GluA2 (Millipore), 1∶2000; GluN2B (Millipore), 1∶1000; GluK2 (Abcam, Cambridge, MA, USA), 1∶1000; vesicular glutamate transporter 2 (vGluT2) (Millipore), 1∶1000; glutamic acid decarboxylase 67 (GAD67) (Millipore), 1∶1000; postsynaptic protein density 95 (PSD95) (Affinity Bioreagents, CO, USA), 1∶2500 and Synaptophysin (Zymed, Carlsbad, CA, USA), 1∶2500 were used and kept overnight for incubation at 4 °C followed by washing and were incubated with horse-radish peroxidase conjugated anti-rabbit or anti-mouse secondary antibody 1∶5000; (Cell Signaling Technology, Danvers, MA, USA) for 1 hr at room temperature followed by washing with TBST. Blots were developed using enhanced chemiluminescent (ECL) Plus Western Blotting Detection System kit RPN2132 (GE Healthcare, Piscataway, NJ, USA) and images were taken using Precision Illuminator Model B95 (Imaging Research Inc., Germany) with a MTI CCD 72S camera and analyzed using MCID Basic software version 7.0 (Imaging Research, St. Catharines, ON, Canada). The X-ray film processor used was model- BMI No 122106 (Brown’s Medical imaging, Omaha, NE, USA). For analysis of protein expression, first, the optical density of each sample was normalized to β-actin. Thereafter, the optical density was normalized to the mean of the WT samples. The average ± SEM of optical densities of GluD1 KO samples, that were normalized to WT mean, are represented as Ratio (KO/WT) ± SEM. The P values were calculated from optical densities of WT and GluD1 KO samples normalized to the WT mean. These data were generated from individual mice and were a representative panel that were repeated several times from independent mice.

### Statistics

Data were analyzed using Student’s unpaired t-test with Welch’s correction or two- way ANOVA (analysis of variance) with Bonferroni’s post-hoc test as necessary for the individual experiment. Differences were considered significant if P≤0.05. Prism 4 (GraphPad Software Inc., San Diego, CA, USA) was used for analysis and representation.

## Results

### GluD1 knockout mice have enhanced working memory and normal reference memory and object recognition memory

**Figure 1:**
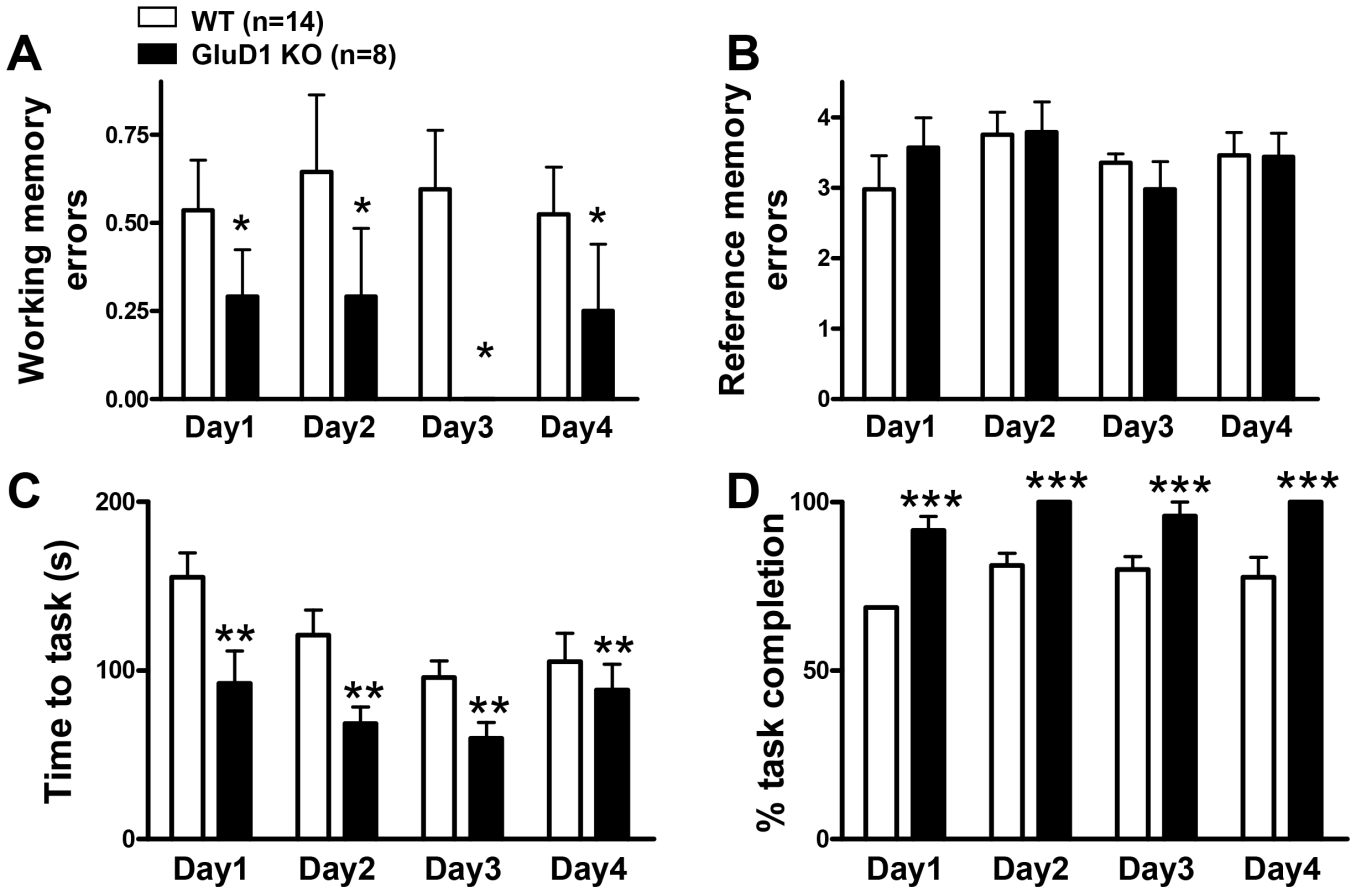
GluD1 KO have enhanced working memory and greater motivation for eight arm radial maze task completion. **A and B.** In the eight arm radial maze GluD1 KO mice (n = 8) made significantly fewer working memory errors compared to WT mice (n = 14) (P = 0.0196) but show no significant difference in the reference memory errors compared to WT mice. **C and D.** GluD1 KO mice also completed the task in shorter duration (P = 0.0051) and completed the task more often than WT mice (P<0.0001). Data are presented as mean ± SEM. *** represents P<0.001, ** represents P<0.01 and * represents P< 0.05.

**Figure 2:**
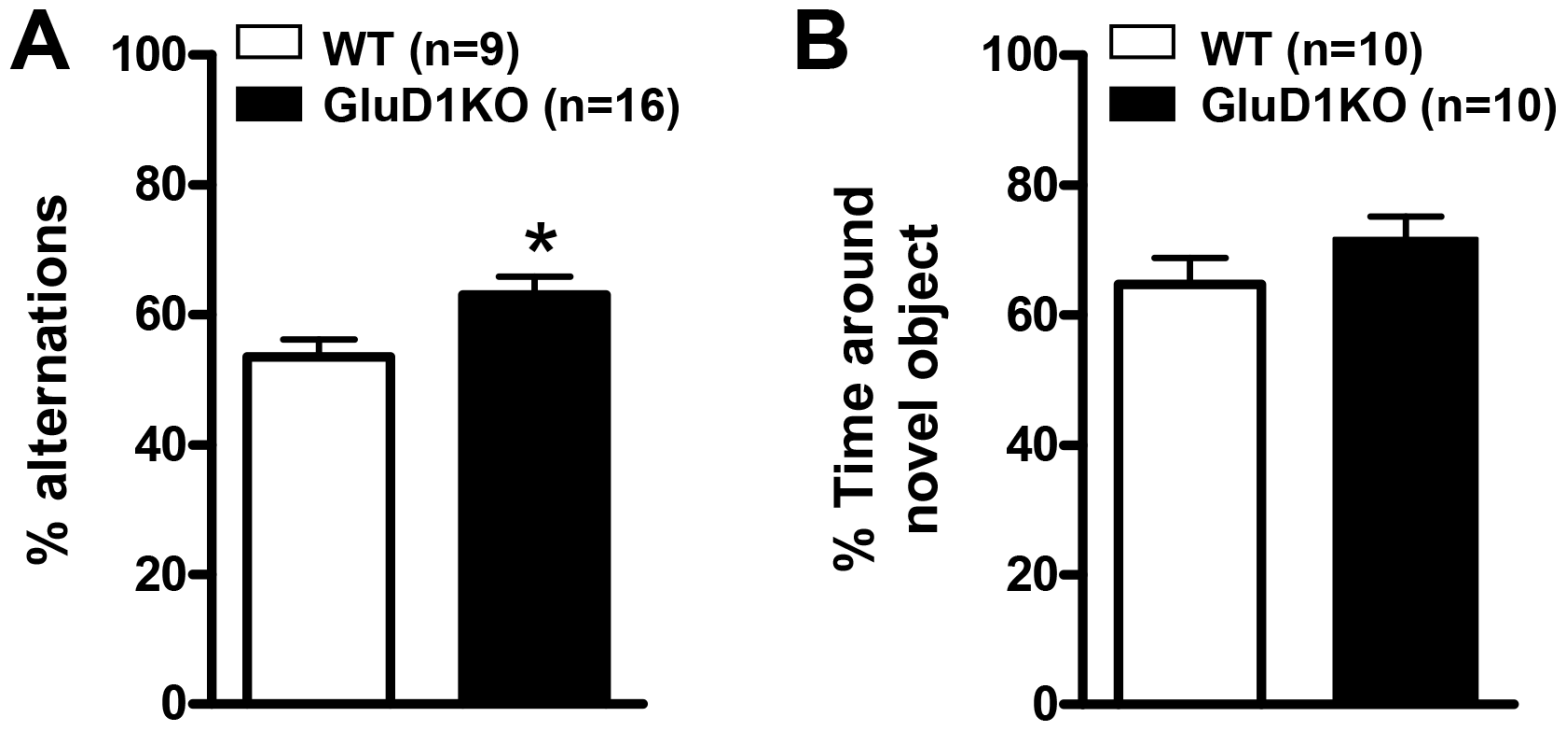
Enhanced working memory in spontaneous Y-maze alternation test in GluD1 KO mice. **A.** GluD1 KO (n = 16) and WT mice (n = 9) were tested in the Y-maze. GluD1 KO were found to show significantly higher percent alternation compared to WT mice (P = 0.0348) suggesting enhanced working memory in GluD1 KO. **B.** GluD1 KO mice (n = 10) behaved similar to WT mice (n = 10) in the object recognition test for reference memory (P = 0.1975). Data are presented as mean ± SEM. * represents P< 0.05.

The eight arm radial maze is a measure for spatial learning and memory in mice [25] that measures both long-term reference memory and short-term working memory**.** In the food motivated eight arm radial maze GluD1 KO mice (n = 8) were similar to the WT mice (n = 14) in reference memory errors (RME) but made significantly fewer working memory errors (WME) (Fig. 1; two-way ANOVA for RME interaction [F(1,60) = 0.4658, P = 0.7072]; testing day [F(1,60) = 0.5789, P = 0.6311]; genotype [F(1,60) = 0.0051, P = 0.9435]. Two-way ANOVA for WME interaction [F(1,60) = 0.6135, P = 0.6089]; testing day [F(1,60) = 0.2065, P = 0.8915]; genotype [F(1,60) = 6.433, P = 0.0196]). GluD1 KO also took shorter time for task completion and had greater percent task completion although these characteristics may be confounded by hyperactivity in GluD1 KO (Fig. 1; two-way ANOVA for time to task interaction [F(1,60) = 0.9379, P = 0.4281], testing day [F(1,60) = 3.574, P = 0.0190], and genotype [F(1,60) = 9.895, P = 0.0051]. Two-way ANOVA for percent task completion interaction [F(1,60) = 0.3364, P = 0.7993], testing day [F(1,60) = 2.649, P = 0.0965], and genotype [F(1,60) = 1560, P<0.0001]). It should be noted that GluD1 KO mice appeared to follow a patterned movement whereby they entered the next adjacent arm each time which may lead to lower working memory errors. We next tested GluD1 KO mice in Y-maze alternation test which is a spatial working memory task based on the natural tendency of the mice to alternate between the three arms. GluD1 KO (n = 16) showed significantly higher percent alternations compared to WT (n = 9) (Fig. 2A; unpaired t-test P = 0.0348, F = 1.909) suggesting enhanced working memory in GluD1 KO. We further tested GluD1 KO mice in the object recognition test for reference memory. GluD1 KO mice (n = 10) displayed learning abilities equivalent to the WT mice (n = 10) (Fig. 2B; unpaired t-test P = 0.1975, F = 1.475).

### GluD1 knockout mice have deficit in reversal learning in the Morris water maze test

**Figure 3:**
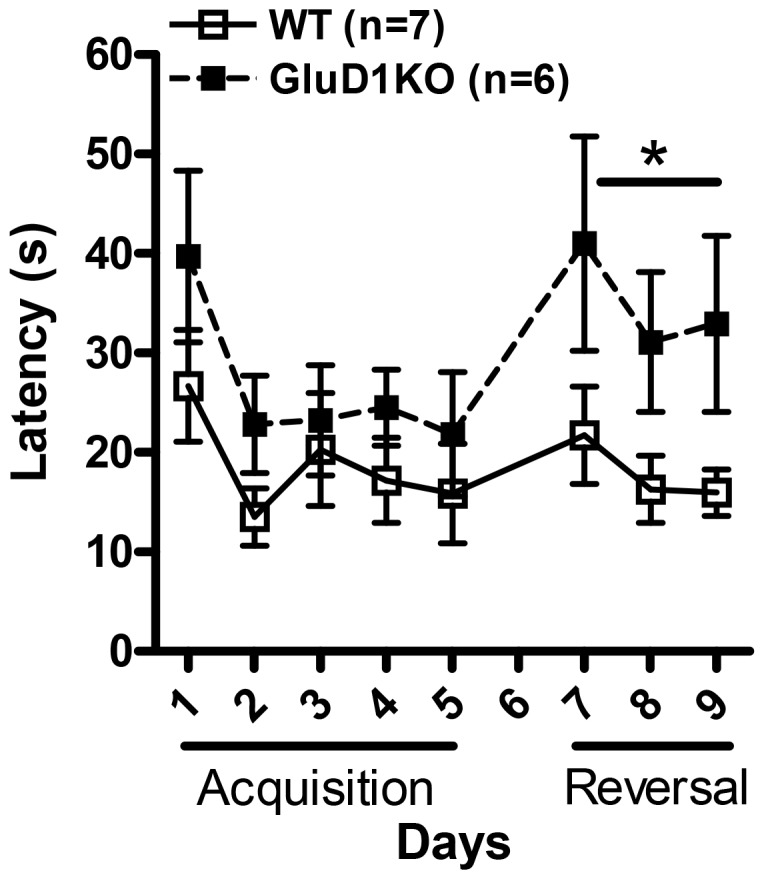
Deficit in reversal learning in the Morris water maze in GluD1 KO mice. Mice were trained in the Morris water maze test. No difference was observed between GluD1 KO (n = 6) and WT (n = 7) mice in the latency to find the hidden platform during the acquisition phase that lasted over 5 days. During the reversal phase GluD1 KO mice took significantly longer time to find the hidden platform in the new location suggesting impaired reversal learning (P = 0.0485). Data are presented as mean ± SEM. * represents P< 0.05.

Previous study on GluD1 KO has indicated that they have normal latency to find the hidden platform in the Morris water maze test [2], however it is not known whether reversal learning in a spatial memory task is affected by deletion of GluD1. We conducted a 5 day acquisition training in the Morris water maze followed by a 3 day reversal task with an interval of 48 hrs between the acquisition and reversal training during which a probe trial was conducted. Similar to a previous study [2] we did not find any significant difference in the latency to find the platform between the GluD1 KO (n = 6) and WT (n = 7) during the acquisition phase (Fig. 3; two-way ANOVA for interaction [F(1,44) = 0.3195, P = 0.8634]; testing day [F(1,44) = 3.352, P = 0.0176]; genotype [F(1,44) = 2.666, P = 0.1308]). During the reversal phase we found that the GluD1 KO mice consistently required a significantly longer time to find the platform placed in a different quadrant than the original location in comparison to WT mice suggesting impairment in the reversal learning task (Fig. 3; two-way ANOVA for interaction [F(1,22) = 0.1301, P = 0.8787]; testing day [F(1,22) = 1.844, P = 0.1817]; genotype [F(1,22) = 4.920, P = 0.0485]).

### GluD1 knockout mice manifest deficits in contextual fear learning

**Figure 4:**
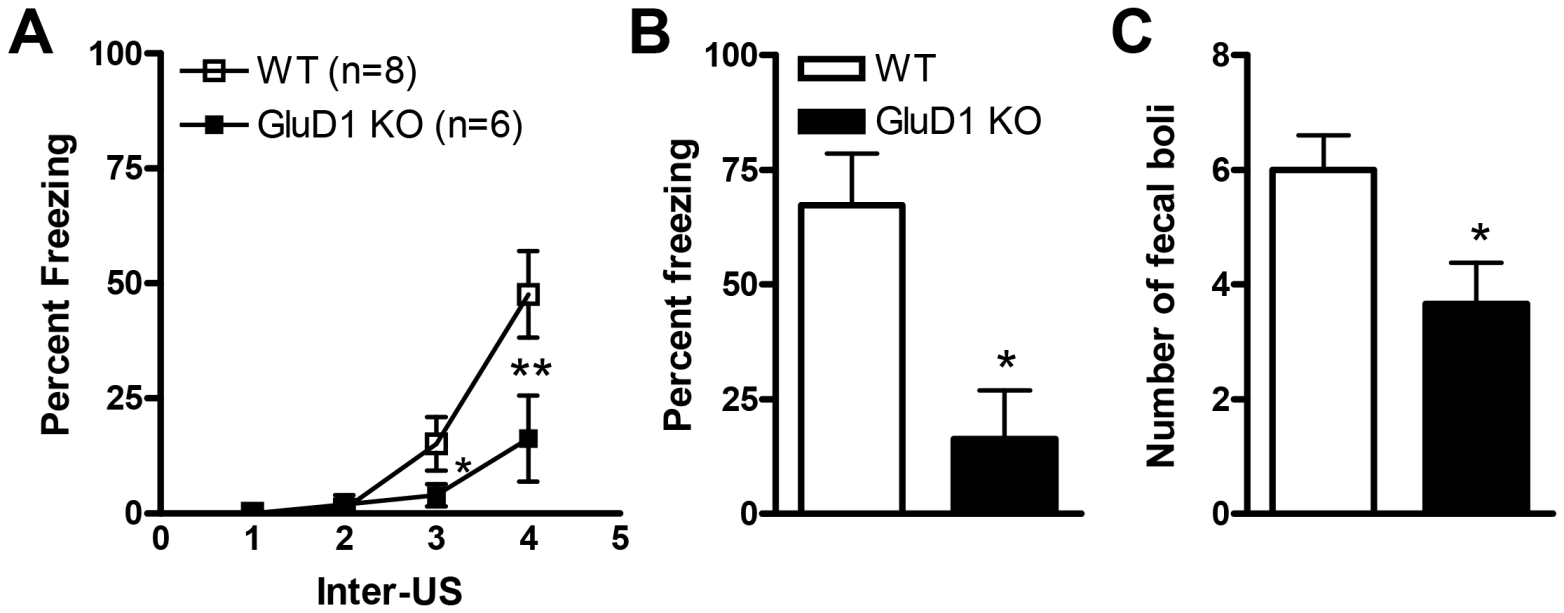
GluD1 KO mice manifest deficits in contextual fear learning. Significant deficit in fear acquisition is observed in GluD1 KO mice (n = 6) during the fourth US session compared to WT mice (n = 8) (P = 0.0400). 24 hrs later after fear conditioning, in the context post-test, the freezing response was significantly lower in GluD1 KO mice versus WT mice (P = 0.0113). Fecal boli produced were significantly lesser for the GluD1 KO mice versus WT mice (P = 0.0108). Data are presented as mean ± SEM. * represents P< 0.05.

As mentioned previously GluD1 is highly expressed in the hippocampus [16]. Thus we performed contextual fear conditioning on GluD1 KO and WT mice to assess the effect of ablation of GluD1 on hippocampus-dependent fear learning. Significantly lower freezing was observed during acquisition in GluD1 KO mice (n = 6) relative to WT mice (n = 8) (Fig. 4A, two-way repeated measures ANOVA; interaction [F(3,33) = 4.92, P = 0.0062], genotype [F(1,11) = 4.74, P = 0.052] and US presentation [F(3,33) = 18.90, P<0.0001] with Bonferroni post hoc test showing significant difference between genotypes after the 3^rd^ presentation of the US (P<0.001)). Twenty-four hrs after conditioning animals were placed back into the conditioning context and freezing behavior was monitored. GluD1 KO mice manifested significantly lower freezing relative to WT mice during testing (Fig. 4B; unpaired t-test, P = 0.0079, F = 1.327). Since freezing behavior may be confounded by hyperactivity in GluD1 KO we also counted the number of fecal boli produced during the context post-test. The number of fecal boli produced was significantly less for the GluD1 KO (n = 6) mice compared to the WT mice (n = 8) (Fig. 4C; unpaired t-test, P =  0.0108, F = 1.377).

### GluD1 knockout mice manifest deficits in cue fear learning

#### Light cue fear learning

**Figure 5:**
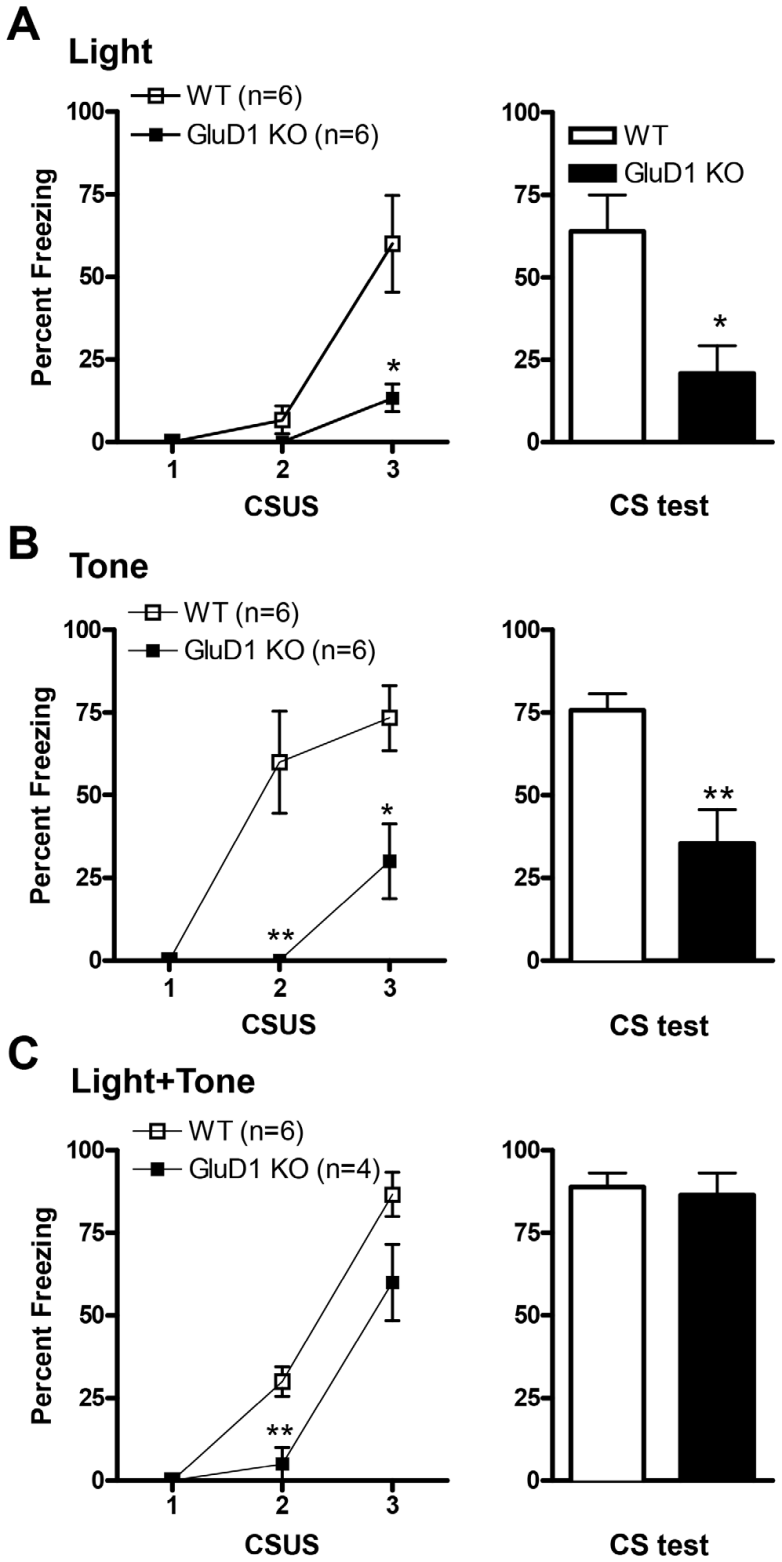
GluD1 KO mice manifest deficits in cue fear learning. **A.** Significant deficit in light fear acquisition is observed in GluD1 KO mice (n = 6) during the third CS-US session compared to WT mice (n = 6) (P = 0.0118). 24 hrs after fear conditioning, in the light CS post-test GluD1 KO manifested significantly lower freezing response compared to WT mice (P = 0.0135). **B.** Significant deficit in tone fear acquisition is observed in GluD1 KO mice (n = 6) during the second and third pairing of the CS and US compared to WT mice (n = 6) (third CS P = 0.0178). In the tone CS post-test, GluD1 KO mice manifest a significant deficit in the freezing versus WT mice (P = 0.0093). **C.** Significant deficit in fear acquisition is observed in GluD1 KO mice (n = 4) during the second CS and US presentation when the CS is a tone + light cue compared to WT mice (n = 6) (P = 0.0074). In the tone + light CS post-test, no significant difference was observed between GluD1 KO (n = 4) mice compared to WT mice (n = 6) (P = 0.7711). Data are presented as mean ± SEM. ** represents P<0.01 and * represents P< 0.05.

We next tested cue fear conditioning in GluD1 KO. GluD1 KO (n = 6) showed significantly lower freezing during the acquisition of light cue conditioning compared to WT mice (n = 6). (Fig. 5A, two-way repeated measures ANOVA; interaction [F(2,20) = 7.68, P = 0.0034], genotype [F(1,10) = 11.43, P = 0.007], and CS presentation [F(2,20) = 19.82, P<0.0001] with Bonferroni post hoc test showing significance at the 3^rd^ presentation of CS (P<0.001)). Twenty-four hrs after conditioning animals were placed in a novel context and freezing behavior to the CS (light) was recorded. Similar to conditioning, GluD1 KO mice exhibited significantly lower freezing in response to the CS versus WT counterpart (Fig. 5A, unpaired t-test; P = 0.0135, F = 1.753).

#### Tone alone fear learning

To further ascertain that cue fear conditioning was impaired in GluD1 KO we used a different cue, tone, in the following experiment. In this test, mice were presented with three pairings of a tone (CS) co-terminating with foot-shock (US). Freezing response was recorded during the duration of tone delivery. GluD1 KO mice (n = 6) showed significantly lower freezing relative to WT mice (n = 6) during the second and third tone delivery during acquisition phase. (Fig. 5B, two-way repeated measures ANOVA; interaction [F(2,20) = 6.78, P = 0.0057], genotype [F(1,10) = 19.61, P = 0.0013] and CS presentation [F(2,20) = 19.03, P<0.0001] with Bonferroni post hoc test showing significance between genotypes at CS2 (P<0.001) and CS3 (P<0.01)). The tone CS post-test was conducted in a novel environment where the CS was a tone. Once again GluD1 KO mice displayed significantly lower freezing response relative to WT mice (Fig. 5B; unpaired t-test, P = 0.0053, F = 4.191). GluD1 KO mice have previously been shown to have high frequency hearing deficit [2](> 20 kHz,). However, it should be noted that the frequency of tone used for fear conditioning was 3 kHz, a frequency where GluD1 KO mice display similar response as WT [2] and therefore may not be a confounding factor. Overall, GluD1 KO have a deficit in both contextual and cue fear conditioning.

#### Tone + light cue fear learning

We next tested whether presence of a stronger CS such as a combination of light and tone cue can overcome the deficit in cue fear conditioning since pairing two conditioned stimuli together with the unconditioned stimulus provides for a greater saliency of the stimuli. Thus we tested tone + light cue fear conditioning in GluD1 KO. GluD1 KO (n = 4) showed significantly lower freezing during the acquisition of tone + light cue conditioning compared to WT mice (n = 6) (Fig. 5C, two-way repeated measures ANOVA; interaction [F(2,16) = 3.35, P = 0.0608], genotype [F(1,8) = 13.37, P = 0.0064] and CS presentation [F(2,16) = 88.17, P<0.0001] with Bonferroni post hoc test showing significant difference between genotypes at CS2 (P<0.05) and CS3 (P<0.01)). However in the CS post-test, no significant difference was observed between GluD1 KO (n = 4) mice compared to WT mice (n = 6) (Fig. 5C; unpaired t-test, P = 0.7540, F = 1.636). Thus pairing of two cues appears to rescue deficit in consolidation of fear memory in GluD1 KO.

### D-Cycloserine reverses contextual fear conditioning deficit in GluD1 knockout mice

**Figure 6:**
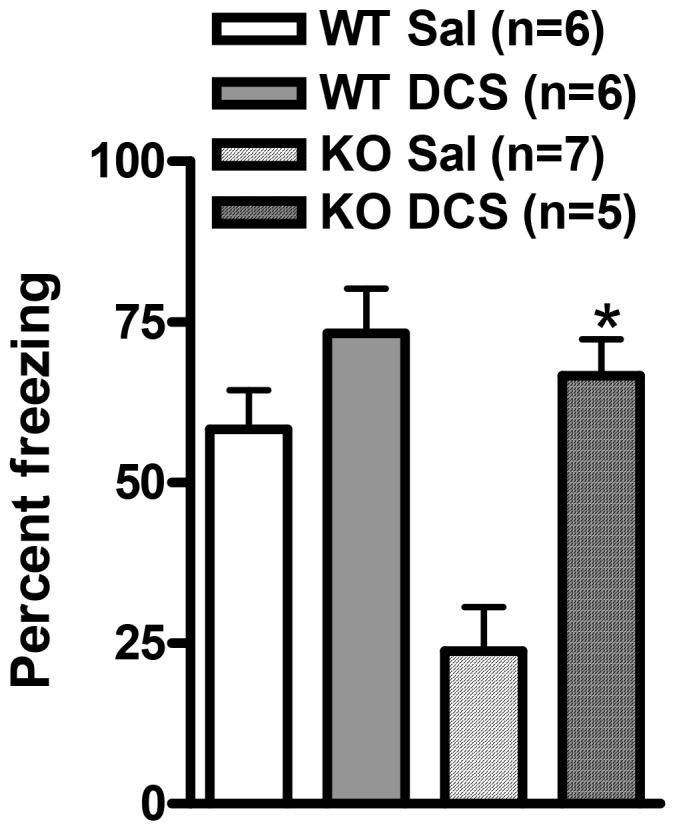
D-Cycloserine (DCS) rescues contextual fear conditioning deficit in GluD1 KO. WT and GluD1 KO mice were intraperitoneally injected with saline or DCS (30 mg/kg) 30 min prior to contextual fear conditioning (n = 5–7 for each group). DCS administration in GluD1 KO significantly enhanced percent freezing during the post-test (two-way ANOVA, drug F(1, 20) = 18.97, P = 0.0003; genotype F(1, 20) = 9.616, P = 0.0056; interaction F(1, 20) = 4.424, P = 0.0483). Data are presented as mean ± SEM. * represents P< 0.05.

D-Cycloserine (DCS) is an NMDA receptor GluN1 glycine-site agonist. We have previously shown that DCS was able to rescue social interaction deficit in GluD1 KO [16]. Due to the remarkable deficit in both cued and contextual fear conditioning in GluD1 KO we sought to determine if DCS administration prior to fear conditioning could rescue fear acquisition deficit. WT and GluD1 KO mice (n = 5–7) were intraperitoneally injected with DCS (30 mg/kg) or saline 30 minutes before contextual fear acquisition. Freezing was monitored in the same context 24 hrs after conditioning. Remarkably, DCS administration in GluD1 KO significantly enhanced percent freezing during the post-test (Fig. 6; two-way ANOVA, drug F(1, 20) = 18.97, P = 0.0003; genotype F(1, 20) = 9.616, P = 0.0056; interaction F(1, 20) = 4.424, P = 0.0483). This data suggests that DCS may rescue deficit in synaptic plasticity required for fear learning in GluD1 KO.

### GluD1 knockout mice have normal latent inhibition and heightened sensitivity to pain

**Figure 7:**
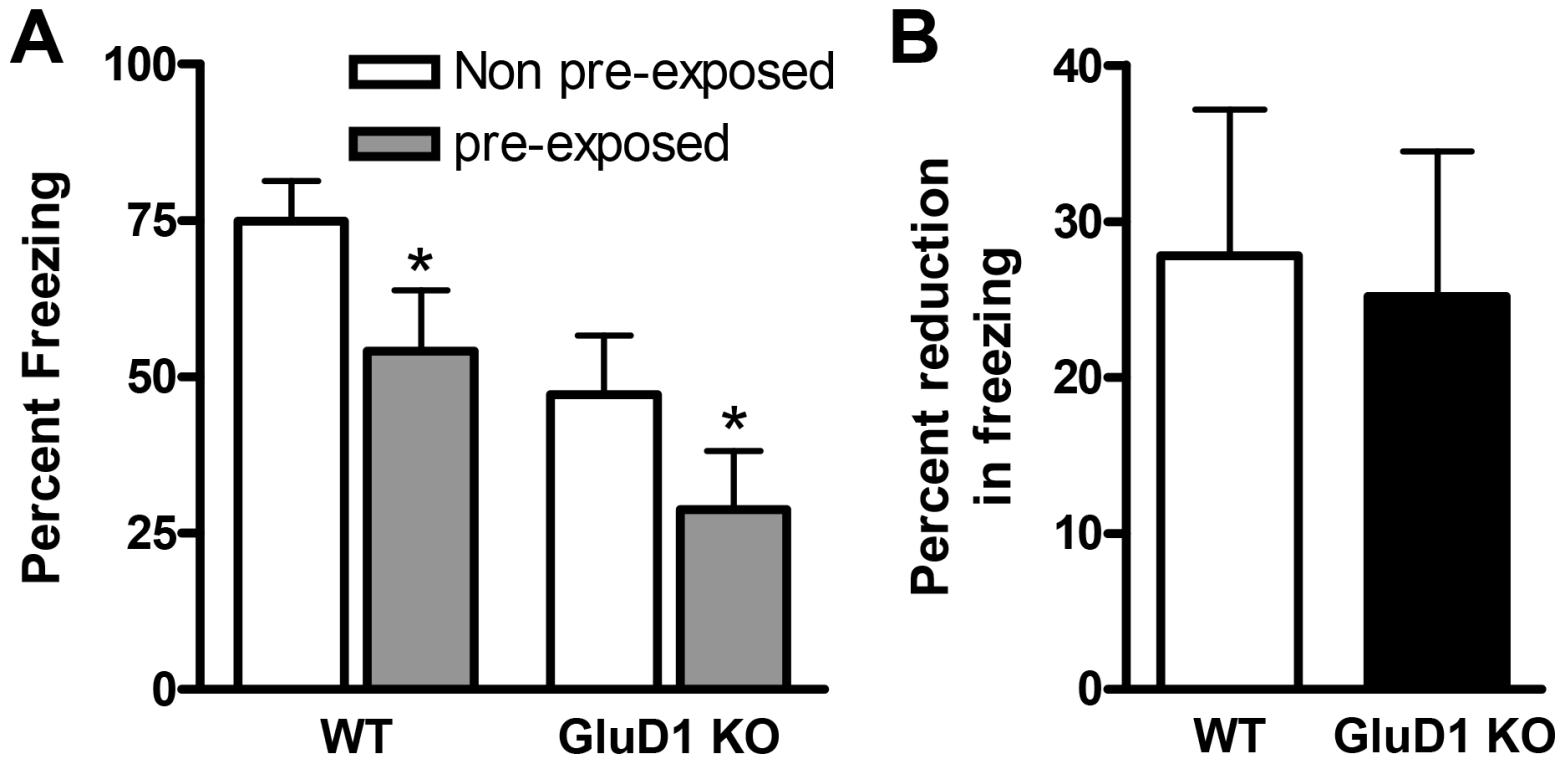
GluD1 KO mice have normal latent inhibition. **A.** There was no significant effect of the genotype X treatment effect between the GluD1 KO and WT mice. However, there was a significant difference between the non-pre-exposed (NPE) and pre-exposed (PE) groups for both the genotypes (n = 11–13 for each group). Additionally there was also a significant difference between the two genotypes in percent freezing (two-way ANOVA interaction P = 0.8928; group P = 0.0342; genotype P = 0.0050). **B.** On further analysis we found no significant difference in the degree of reduction in percent freezing due to pre-exposure between the WT and GluD1 KO mice. Data are presented as mean ± SEM. * represents P< 0.05.

**Figure 8:**
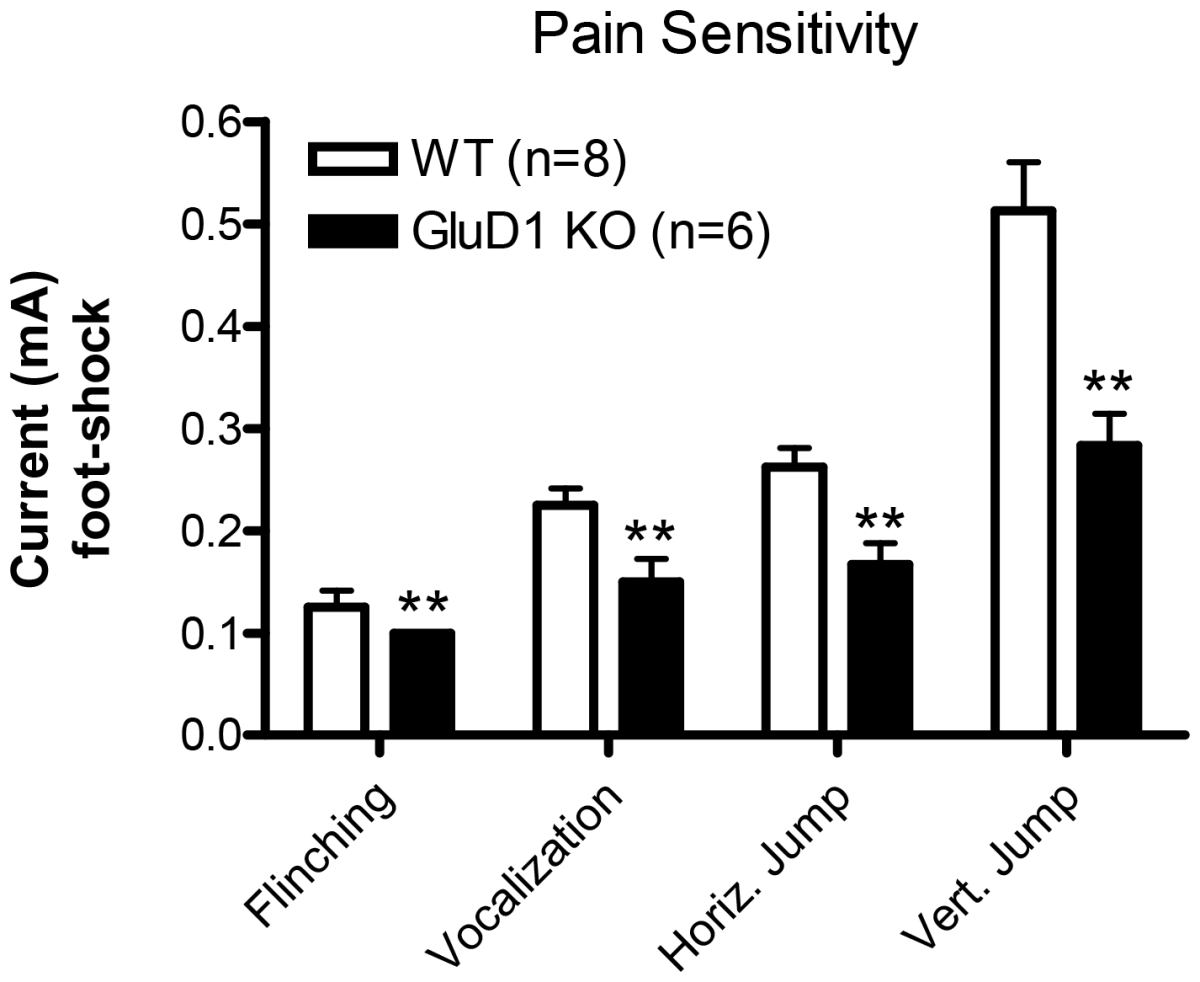
GluD1 KO have higher pain sensitivity to foot-shock. In the test for pain sensitivity GluD1 KO (n = 8) mice had a significantly higher sensitivity to pain elicited by footshock compared to WT mice (n = 6) (two-way ANOVA interaction F(3,48) = 5.340, P = 0.0030; pain response F(3,48) = 41.26, P<0.0001; genotype F(1,43) = 31.74, P<0.0001). Data are presented as mean ± SEM. ** represents P<0.01.

Latent inhibition is a sensorimotor test relevant to schizophrenia-like behavior. Since GRID1 gene has been found to be associated with schizophrenia we tested latent inhibition in GluD1 KO mice. WT and GluD1 KO mice were divided into four groups before initiation of the test, WT non-pre-exposed (NPE), WT pre-exposed (PE), GluD1 KO non-pre-exposed (NPE) and GluD1 KO pre-exposed (PE) (n = 11-13 for each group). As expected pre-exposure to tone reduced freezing in both WT and GluD1 KO mice. Moreover, in agreement with a deficit in tone fear conditioning a significant difference in freezing was observed between the two genotypes. However, there was no significant effect of the genotype X treatment between the GluD1 KO and WT mice (Fig. 7A; two-way ANOVA interaction [F(1,43) = 0.01836, P = 0.8928], group [F(1,43) = 4.783, P = 0.0342], and genotype [F(1,43) = 8.766, P = 0.0050)]). On further analysis we found no significant difference in the percent reduction in freezing due to pre-exposure in WT and GluD1 KO mice (Fig. 7B; unpaired t-test, P = 0.8467, F = 1.109). Thus GluD1 KO appear to be normal in the latent inhibition test. Interestingly, GluD1 KO (n = 8) mice showed significantly higher sensitivity to pain elicited by foot-shock compared to WT mice (n = 6) (Fig. 8; two-way ANOVA interaction [F(3,48) = 5.340, P = 0.0030], behavioral response [F(3,48) = 41.26, P<0.0001], and genotype [F(1,43) = 31.74, P<0.0001]).

### Molecular abnormalities in the hippocampus of GluD1 knockout mice

**Figure 9:**
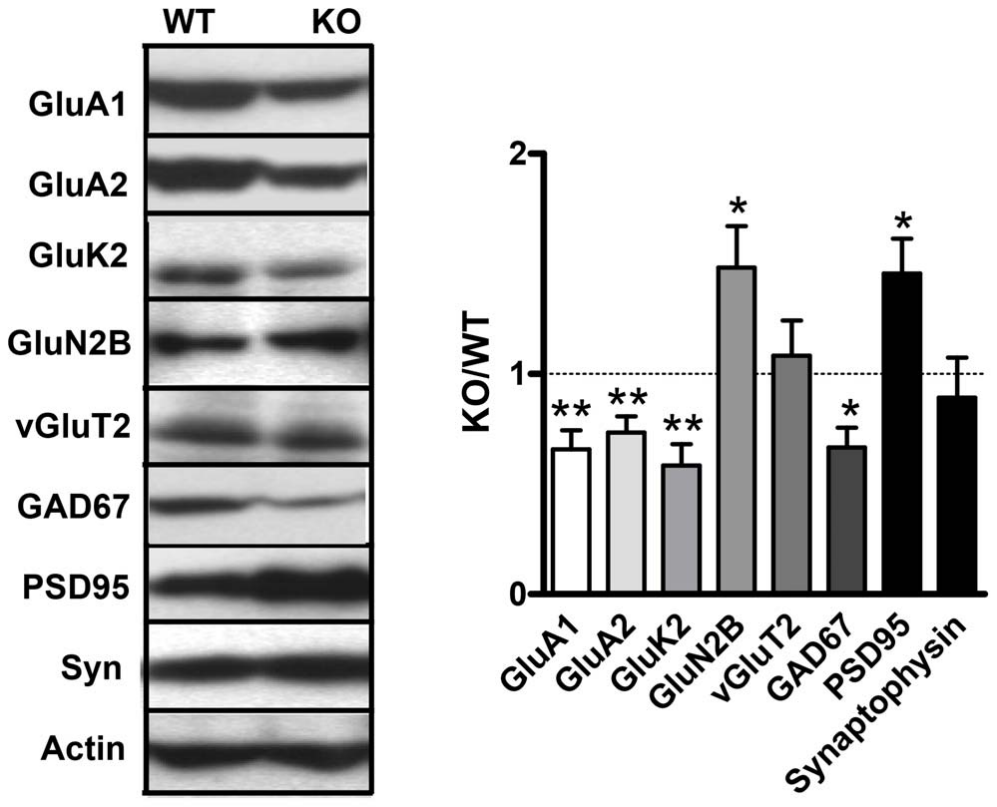
Altered expression of iGluR subunits and synaptic proteins in GluD1 KO hippocampus. In synaptoneurosomal preparations from the hippocampus of GluD1 KO and WT (n = 6-11) a significantly lower expression of GluA1 (P = 0.0030), GluA2 (P = 0.0034), GluK2 (P = 0.0021) and GAD67 (P = 0.0115) while there was a significantly higher expression of GluN2B (P = 0.0328) and PSD95 (P = 0.0272) observed in GluD1 KO. These data were generated from individual mice and were a representative panel that were repeated several times from independent mice. Data are presented as mean ± SEM. ** represents P<0.01 and * represents P< 0.05.

Next we wanted to determine if there were alterations in the expression of iGluR subunits and synaptic proteins in the hippocampus of GluD1 KO versus WT mice which may lead to the learning and memory deficits reported here. In our previous study we examined the expression of iGluR subunits and synaptic proteins in the prefrontal cortex and amygdala of GluD1 KO mice. We performed similar analysis in the hippocampus. We examined the effect of GluD1 deletion on proteins representative of; (1) iGluR subunits: GluA1, GluA2, GluK2, GluN2B; (2) presynaptic and postsynaptic proteins: synaptophysin and PSD95 respectively and (3) excitatory and inhibitory neurons: vGluT2 and GAD67 respectively. Examining the GluD1 KO to WT ratio (n = 6–11) in the hippocampus we found that there is a significantly lower expression of GluA1 (P = 0.0030), GluA2 (P = 0.0034), GluK2 (P = 0.0021) and GAD67 (P = 0.0115) and a significantly higher expression of GluN2B (P = 0.0328) and PSD95 (P = 0.0272) in the GluD1 KO brain versus the WT brain (Fig. 9). These results suggest that deletion of GluD1 leads to changes in the expression of synaptic proteins in the hippocampus.

## Discussion

In the current study we have identified that deletion of GluD1 leads to unique abnormalities in learning and memory. We found enhanced working memory in the eight-arm radial maze and spontaneous Y-maze alternation test, deficit in reversal learning in Morris water maze and deficit in contextual and cued fear conditioning in GluD1 knockout mice. We also found that deletion of GluD1 leads to molecular abnormalities in the hippocampus which may be responsible for some of the behavioral alterations in GluD1 knockout mice

### Specific learning deficits in GluD1 knockout

In the eight-arm radial maze test GluD1 knockout mice manifested an enhanced working memory, lower time for task completion and greater percent task completion (Fig. 1). Enhanced working memory was also observed in the spontaneous Y-maze alternation test (Fig. 2A). Working memory is an immediate and rapidly decaying memory sustained by plasticity in the prefrontal cortex–hippocampus network [26], [27], [28], [29], [30], [31]. Thus altered plasticity or synaptic efficacy mechanisms in prefrontal cortex or hippocampal synapses may underlie the observed enhanced working memory in GluD1 knockout. This hypothesis will require further direct testing using functional assays. It should be noted that repetitive or stereotyped behavior as evident by enhanced marble burying [32] observed in the GluD1 knockout mice along with hyperlocomotion [16], may partly be responsible for the lower working memory errors in the radial maze and greater alternations in the Y-maze. Another unique abnormality in GluD1 knockout was a deficit in reversal learning in the Morris water maze test (Fig. 3). GluD1 knockout showed similar latency during acquisition training but spent longer time to find the hidden platform during reversal learning. Reversal learning requires plasticity mechanisms most likely involving long-term depression (LTD) in the hippocampus [22], [33]. Deletion of GluD1 does not affect long-term potentiation (LTP) [2], however, its effect on LTD is not known.

GluD1 knockout mice manifested both contextual and cue fear conditioning deficits (Fig. 4, 5). Fear acquisition and anxiety like behaviors share a common neural circuitry with amygdala being the site that regulates these behaviors [34], [35]. In addition a role of hippocampus has been demonstrated in contextual fear conditioning [36], [37], [38]. The deficit in fear acquisition in GluD1 knockout is therefore in agreement with our previous findings showing that GluD1 knockout exhibit lower anxiety-like behavior in the plus maze test and abnormalities in expression of synaptic proteins in the amygdala and hippocampus [16]. Furthermore, the deficit in contextual fear acquisition was rescued by DCS administration. DCS is known to bring about restoration of synaptic plasticity and learning including contextual fear conditioning and such mechanisms may underlie DCS effects in GluD1 knockout [17]. We have previously found that DCS rescues social deficits in GluD1 knockout mice [16] suggesting that NMDA receptor-dependent mechanisms are sufficient to reverse a range of behavioral deficits.

It is intriguing that GluD1 knockout mice exhibit deficit in reversal and associative fear learning together with enhanced working memory. Similar contrasting effects on learning and memory paradigm have been observed in other knockout models for example Shank 1 and Neuroligin-3 knockout mice [39], [40] and alternation between enhancement and deficits in learning and memory is a feature bearing relevance to certain mental disorders [41], [42]. It is likely that the brain region and cell-type specific molecular abnormalities may lead to these unique learning and memory characteristics in GluD1 knockout mice.

### Molecular abnormalities in GluD1 knockout and relevance to cognitive deficits and behavioral abnormalities

Synaptic defects most likely underlie the observed behavioral deficits in GluD1 knockout. Although the precise nature of these synaptic abnormalities remains to be determined, hippocampal synaptoneurosomes in GluD1 knockout mice show a lower expression of GluA1, GluA2, GluK2 and GAD67. In contrast there was a higher expression of the GluN2B subunit and PSD95 (Fig. 9). We have previously shown in GluD1 knockout a reduced expression of GluA1 and GluA2 in the prefrontal cortex and a higher expression of GluA1 and GluK2 in the amygdala [16]. Thus taken together there appears to be an inverse relationship in the molecular abnormalities in the amygdala and prefrontal cortex while the abnormalities in hippocampus appear to mirror those in the prefrontal cortex.

Hippocampal AMPA and kainate receptors have been implicated in regulating spatial memory, fear acquisition as well as other forms of learning and memory [43], [44], [45], [46], [47], [48]. Alterations in the expression of AMPA and kainate subunits in the hippocampus, prefrontal cortex and amygdala in GluD1 knockout mice may therefore partially explain the altered spatial memory and fear learning seen in GluD1 knockout mice. Interestingly, overexpression of GluN2B subunit in the rodent forebrain, cortex and hippocampus leads to superior memory in behavioral tasks, including object recognition and enhancement of spatial memory in the Morris water maze [49], [50], [51] and therefore higher GluN2B (Fig 9) may potentially underlie enhanced working memory in GluD1 knockout mice. We also found higher PSD95 expression and lower GAD67 expression in GluD1 knockout mice. Overexpression of PSD95 leads to excitatory synapse development [52] and a reduction of inhibitory synapses [53]. Together these findings suggest a potential imbalance in excitatory and inhibitory synapses in the hippocampus of GluD1 knockout mice [53]. Moreover, reduced GAD67 expression in the GluD1 knockout is in agreement with the previous results in cell culture system where GluD1 knockdown has a more marked effect on formation of inhibitory synapses [54].

### Relevance of GluD1 knockout to models of neuropsychiatric disorders

As mentioned previously, genetic studies have shown a strong association of GRID1 gene with schizophrenia, mood disorders and ASDs. Understanding the relationship of behavioral deficits in GluD1 knockout to these mental disorders may reveal novel molecular mechanisms underlying the behavioral abnormalities in these disorders. We observed a deficit in reversal learning in GluD1 knockout mice that is analogous to resistance to change behavior and inflexibility which is a core symptom in ASDs [55], [56], [57]. Reversal learning deficits also feature in human patients with schizophrenia [58] as well as in mouse models for autism and schizophrenia [41], [59], [60], [61], [62], [63], [64], [65]. Similarly, contextual and cue fear conditioning deficits in GluD1 knockout mice also occur in mice models implicated in ASDs and schizophrenia [61], [62], [66], [67], [68] and may represent impaired conditioned association in schizophrenic and ASD individuals [69], [70], [71], [72]. Interestingly, GluD1 knockout mouse manifested an enhanced working memory. The implication of this finding to mental disorders, especially to schizophrenia which is characterized by working memory deficits is unclear. Nonetheless, enhanced working memory has been observed in a mouse model of ASD [39] and superior working memory and enhanced logic are observed in autistic savants [73], [74], [75], [76] and therefore may represent a useful endophenotype in mouse models of ASD.

Among the GRID1 associated disorders ASD has a very early onset at 2–3 years of age while others such as schizophrenia and bipolar disorder emerge in late teenage years and have been proposed to have a neurodevelopmental etiology. Additionally, a number of genes associated with these disorders are crucial for normal synapse formation, function or signaling [77], [78], [79], [80], [81], [82], [83]. The developmental expression pattern [3] and potential role in synapse formation [54], [84] further supports a relationship between GluD1 dysfunction and neurodevelopmental disorders. Our expression results indicating a potential excitatory-inhibitory imbalance (Fig 9) also support association of GluD1 with ASDs and schizophrenia where hypofunctioning of GABAergic system has been demonstrated [85], [86], [87], [88], [89], [90]. Moreover, changes in expression of AMPA and kainate receptors have also been reported in postmortem brains of individuals suffering from ASDs, schizophrenia, major depression and bipolar disorder [91], [92], [93], [94], [95], [96], [97], [98] which has similarity to abnormal AMPA and kainate receptor expression in hippocampus, prefrontal cortex and amygdala that we found in GluD1 knockout mice.

## Conclusions

Together our behavioral and molecular analysis of GluD1 knockout mice strongly implicate that GluD1 dysregulation may lead to behavioral and cognitive deficits observed in mental disorders. GluD1 associated disorders including ASD, schizophrenia and bipolar disorder have several overlapping negative symptoms and cognitive deficits [56] and may have a common genetic link [99]. This points to the possibility that dysregulation of GluD1 may be a predisposing factor for a specific domain of mental disorders. The learning and memory abnormalities identified due to deletion of GluD1 receptors represent a novel functional paradigm for this receptor. Further studies are required to fully understand a potential relationship between GluD1 and mental disorders and evaluate details of the GluD1-mediated mechanisms in the regulation of synapses and circuits.
